# The Persistence of African Swine Fever Virus in Field-Infected *Ornithodoros erraticus* during the ASF Endemic Period in Portugal

**DOI:** 10.1371/journal.pone.0020383

**Published:** 2011-05-31

**Authors:** Fernando S. Boinas, Anthony J. Wilson, Geoff H. Hutchings, Carlos Martins, Linda J. Dixon

**Affiliations:** 1 Centro de Investigação Interdisciplinar em Medicina Veterinária, Faculdade de Medicina Veterinária da Universidade Técnica de Lisboa, Lisboa, Portugal; 2 Vector-Borne Diseases Programme, Institute for Animal Health, Woking, Surrey, United Kingdom; University of Liverpool, United Kingdom of America

## Abstract

African swine fever (ASF) is an important disease of pigs and outbreaks of ASF have occurred in Europe on multiple occasions. To explore the period for which the European soft tick species *Ornithodoros erraticus* (Acari: Argasidae) is able to act as a reservoir of African swine fever virus (ASFV) after infected hosts are removed, we collected specimens from farms in the provinces of Alentejo and Algarve in Portugal during the endemic period and tested them subsequently using cell culture and experimental infection. We show that ticks from previously infected farms may contain infectious virus for at least five years and three months after the removal of infectious hosts. Furthermore, in two cases infectious virus was successfully isolated from ticks on restocked farms that had not yet suffered a re-emergence of disease. Experimental transmission to pigs was demonstrated in batches tested up to 380 days after an outbreak. These results clarify the epidemiological role of *O. erraticus* ticks in the persistence of ASFV in the field, provide additional evidence to support its role in the re-emergence of a sporadic outbreak of ASF in Portugal in 1999 and suggest that the current quarantine legislation and restocking advice when these ticks are present on the pig farm premises is appropriate.

## Introduction

African swine fever (ASF) is an acute haemorrhagic fever caused by African swine fever virus (ASFV), genus *Asfivirus*, family *Asfarviridae*, which can cause mortality approaching 100% in domestic pigs. It is endemic in many Sub-Saharan countries in Africa. The first spread of ASF outside Africa was to Portugal in 1957 as a result of waste from airline flights being fed to pigs near Lisbon airport, and since 1957 outbreaks of ASF have occurred in many parts of mainland Europe including Portugal, Spain, Italy, France, Belgium and the Netherlands. With the exception of Sardinia, Europe is currently considered free of the disease. However, in 2007 a major outbreak of ASF began in Georgia [1]. Nearly 90,000 pigs died in the first six months of this outbreak, and at the time of writing the outbreak has spread to affect Armenia, Azerbaijan and the Russian Federation. As this outbreak clearly illustrates, ASF can have devastating effects on pig production and future outbreaks of ASF in Europe could have significant consequences for food security and animal welfare [2].

ASFV can be transmitted between suid hosts by infected tissues and animal products such as cured ham, by bodily fluids such as blood, and by soft ticks of the genus *Ornithodoros*. At least one European species of *Ornithodoros*, *O. erraticus* (Acari: Argasidae), is known to be capable of ASFV transmission [3]. Although Portugal was officially declared free from ASFV in 1993 [4], the virus re-emerged on a single farm in the province of Alentejo in November 1999 [5]. This outbreak was controlled without spread to other holdings [6]. *Ornithodoros* ticks are long-lived, and it has been suggested that ASFV might be able to persist in European *Ornithodoros* species for up to eight years [7], although the longest period for which evidence of persistence has previously been published is 655 days [8]. ASFV-infected *O. erraticus* surviving from 1993 have therefore been proposed as a potential source of the 1999 Portugal outbreak [6].

Previous studies have explored the persistence of ASFV in *O. erraticus* following experimental infection, and the persistence of DNA in field-infected ticks, but not the long-term persistence of infectious virus in ticks infected under field conditions. Here, we report on the detection of ASFV in *O. erraticus* collected from farms after the infectious pig population had been removed, and the experimental transmissibility of these persistent infections, in order to help understand the potential for this European species to act as a reservoir of ASFV.

## Results

### Collection of *O. erraticus*

*O. erraticus* were collected from 34 farms. A total of 88 collections were made, which yielded 3,443 live *O. erraticus* (85 collections in total) and 128 dead *O. erraticus* (3 collections). One hundred and sixty one further ticks died after collection before they could be tested.

### Experimental transmission

Thirty-eight collections, consisting of a total of 1,662 live ticks, were tested for transmissible infection by experimental feeding on pigs. Experimental feeding was carried out 228-2030 days after the last possible exposure of the ticks to ASFV on the farm. Transmission was observed with four batches, which were collected from farm 108 and tested between 228 and 380 days after the end of the ASF outbreak on the farm (Table 1).

**Table 1:** Results of experimental feeding and cell culture.

Farm	County of origin	Days between outbreak and feeding	# ticks feeding	Transmission	Days between outbreak and cell culture^1^	Isolation
103	Castro Verde	805	24	N	763	Y
		experimental infection by feeding not done			825	N
		867	15	N	937	Y
106	Ourique	experimental infection by feeding not done			1920	Y
		1038	12	N	1962	N
		experimental infection by feeding not done			1835	N
		1178	4	N	1920	N
		1316	51	N	1258	N
		2030	8	N	2060	N
		experimental infection by feeding not done			2927	N
108	Ourique	experimental infection by feeding not done			956	Y
		228	104	Y	956	Y
		228	255	Y	1100	Y
		278	202	Y	1094	N
		380	114	Y	1052	N
		1162	267	N	1052	N
		1162	13	N	1128	N
		experimental infection by feeding not done			1192	N
		experimental infection by feeding not done			2059	N
		experimental infection by feeding not done			1961	N
		1711	5	N	1964	N
		experimental infection by feeding not done			1758	N

Only farms with positive results shown.

1 Time reported is to the date on which the ticks in the batch were frozen for testing, not the date of the test itself.

### ASFV detection in cell culture

**Figure 1:**
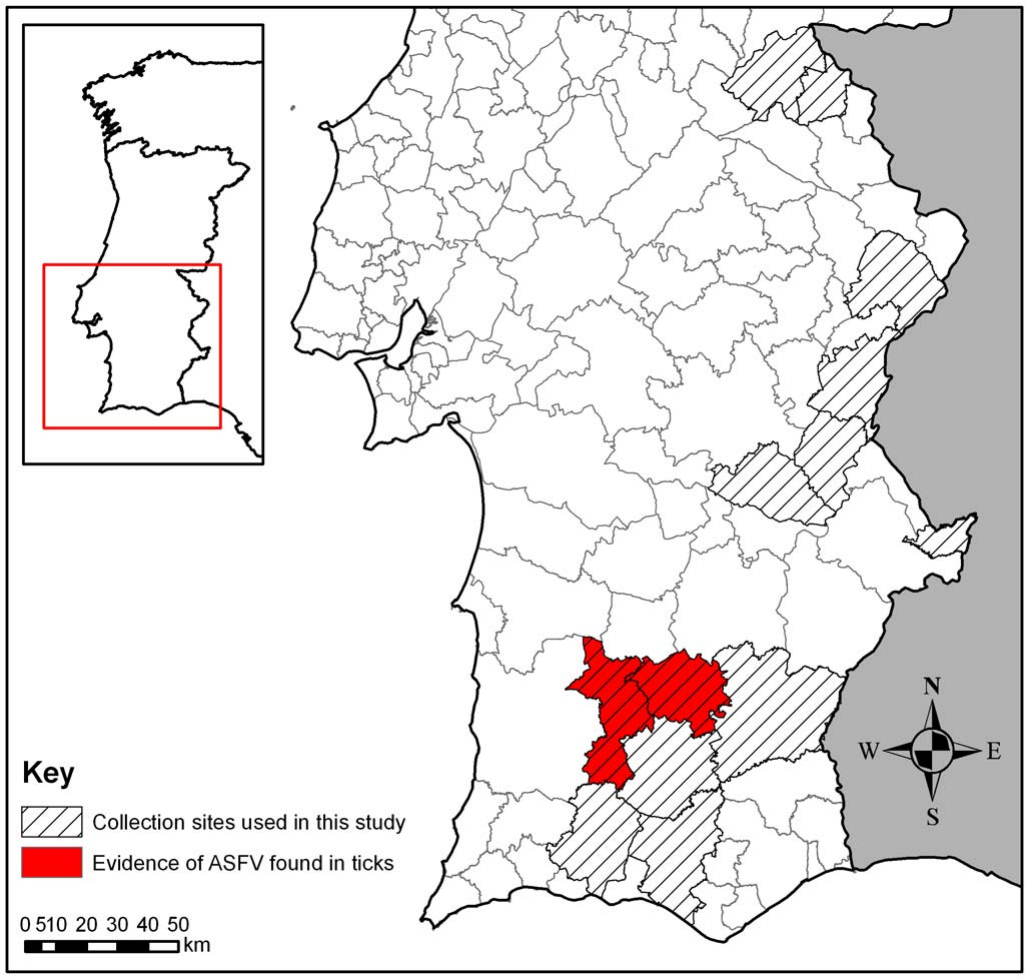
*O. erraticus* collection sites and evidence of ASFV infection.

All surviving *O. erraticus* (3,282 individuals) were tested for replicating ASFV using cell culture. ASFV was detected in four adult ticks via cell culture alone and from six adult ticks via a combination of cell culture and either DIF or PCR. Virus was detected in batches from three of the 34 farms on which collections were made (farms #103, #106 and #108), i.e. 8.8% of the tested farms. These farms were visited three, seven and 11 times respectively, over a period of nearly five years from 1988 to 1993. All three of these farms were in the province of Alentejo (Figure 1). The maximum period between the end of the ASFV outbreak on a farm and the successful isolation of ASFV from collected ticks by cell culture ranged from 937 to 1920 days (Table 1). ). All tick batches resulting in virus transmission to pigs were fully tested and isolation was achieved in two batches within 845/852 days after feeding while no isolation was achieved on batches tested 672 and 816 days after infecting pigs.

## Discussion

Of the 34 farms surveyed in this study, infectious ASFV was detected in ticks collected from three, representing 8.8% of the total, by cell culture and from one of these also by experimental transmission. The three farms from which virus was detected were located in two neighbouring counties, Ourique and Castro Verde, in the southern part of Alentejo (Figure 1). Infected ticks were found only on farms which had either previously reported an outbreak of clinical ASF or on which seropositive animals were present. There are no previous reports on the farm-level prevalence of ASFV infection in *O. erraticus* populations in the field in Portugal, but in a survey carried out from 1987 to 1989 in Spain, ASFV-infected ticks were found on 6% of surveyed farms in the provinces of Salamanca and Huelva while no virus was isolated from ticks collected on farms in the Province of Badajoz (Sanchez-Vizcaino, J. M., personal communication).

Under current EU legislation, buildings on pig farms can be restocked as soon as 40 days after an ASF outbreak in the absence of vectors, following cleaning and disinfection, but where vectors are believed to be involved in transmission the minimum quarantine period is extended to six years [19]. In this study, experimental transmission was demonstrated from ticks collected over a year after the end of the outbreak, and infectious virus was isolated from ticks collected more than two years after an outbreak and maintained in the laboratory for a further three years. These results confirm that *O. erraticus* can transmit ASFV to pigs for over a year after the removal of infectious hosts and suggest that infectious virus can persist in tick populations for at least five years, indicating that the current quarantine period of six years is appropriate for areas where *Ornithodoros* ticks are known or suspected to occur.

Current FAO guidance suggests that restocking should initially occur at 10% of the original density and the animals then monitored for six weeks for clinical signs of ASFV before restocking occurs [20]. On two of the three farms in this study where infectious virus was detected in ticks, pigs present on the farm for up to a year had not become infected. Although experimental transmission from these ticks was not demonstrated, this result indicates that sentinel pigs are of limited effectiveness for detecting ASFV in the tick population.

The last tick collection in this study from which infectious ASFV was detected via cell culture was made more than 4 months after the end of the endemic period of ASF in Portugal. A single outbreak occurred in the same province in 1999. The results of this study suggest that persistence in field populations of *O. erraticus* could have been responsible for this late and brief re-emergence. In Spain the last provinces to eradicate ASF were also the ones with *O. erraticus* presence [21], which is likely to have complicated eradication.

Our study adds considerably to the evidence that the European species *O. erraticus* represents a reservoir for ASFV in the medium and long term. It is known that soft ticks are able to be without feeding for periods of up to 5 years in the cases of large nynphs and adults [10], [22]. The life span of soft ticks can be of up to 15–20 years [23] and they can feed on alternative hoststo pigs, such as sheep and goats, rabbits, chickens and birds[10], [24], [25].

Portugal has been free of ASF since the single reoccurrence of 1999, and tighter quality control measures on meat during the last 10–15 years have placed increased economic pressure on pig farmers in Alentejo to protect their animals from tick bites in order to reduce the frequency of subcutaneous haemorrhages and haematomas. A national campaign to inform farmers about the involvement of ticks in the transmission of ASFV has also been conducted by the Portuguese veterinary services, and in both Portugal and Spain the re-use of premises with established tick populations following an outbreak has been restricted [21]. As a result, some traditional pigsties have been abandoned or destroyed, probably reducing the number of farms on which *O. erraticus* could act as a reservoir of ASFV in the event of a future outbreak. At the same time, the production of Alentejano pigs using traditional extensive methods has grown in recent years [26], increasing the risk that farmers may be tempted to reuse abandoned buildings infested by ticks in the future. Furthermore, although the European distribution of *O. erraticus* is limited to Portugal and Spain, other members of the species complex occur in parts of southern Europe, North Africa and the Caucasus [2]. Given the economic importance of ASFV and the extent to which *Ornithodoros* species can complicate its eradication, the potential role of these related *Ornithodoros* species in the epidemiology of ASFV must be clarified.

## Materials and Methods

### Ethics statement

The experiments on pigs described below were carried out under Home Office Licence 90/00752. Although all experimental protocols on project licences were formally approved by the Home Office Inspector, the experiments were carried out prior to the requirement for an Ethical Review Committee. The experimental design was retrospectively reviewed by the current Ethics Committee for Laboratory Animals at FMV on the 7th of February 2011, who indicated that in their opinion all ethical procedures were designed according to good animal practices with respect to welfare, and were carried out according to the Council Directive regarding the protection of animals used for experimental and other scientific purposes (6/609/EEC), and National Legislation.

### Collection, identification and maintenance of *O. erraticus*

Thirty-four pig farms in Alentejo and Algarve were selected for study based on a survey of local veterinary knowledge of tick presence [9]. Buildings used to house pigs on these premises were sampled for *O. erraticus* by removing and examining soil and the contents of cracks and crevices in the walls, roof tiles and ground. The earth and dust collected by the manual method were screened for ticks [10]. During warmer months (spring, summer, autumn) and when it was possible to exclude pigs from the building to avoid trap damage, a CO_2_-baited trap [11] was also used. The collected ticks were sorted by developmental stage and separated into batches of various sizes (8–10 individuals per pool for larvae and stage 1 nymphs, 6–8 individuals for stage 2 or stage 3 nymphs, 5–6 individuals for stage 4 or stage 5 nymphs and 2–4 specimens for adults). Batches which were to be fed on pigs were kept in plastic containers covered with a fine nylon cloth (16 mesh cm^−1^) and maintained in incubators at 27–28°C and 85% relative humidity. Other batches were killed by freezing at ∼80°C until required for testing.

### Experimental transmission to pigs

Batches of live ticks were first allowed to feed on susceptible pigs when these were available. Susceptible crossbred Large White/Landrace pigs of 30–40 kg weight were anaesthetised with Pentobarbitone-Na (Sagatal^R^, May & Baker Ltd) and pots of ticks were placed against a shaved area of the abdomen and held there with adhesive tape. Containers were removed after 20–30 minutes or until the ticks had engorged and detached. Between four and 453 individuals were allowed to feed on each individual pig. After removal, ticks were anesthetised using CO_2_ and engorged and unengorged ticks were sorted into separate containers. Post-feeding, clinical examination and rectal temperatures were recorded each day. Viraemia and antibodies were monitored weekly or when the temperature of pigs rose above 39.5°C. This was carried out until the animals died or were euthanized or for a minimum period of 3 weeks.

Blood samples were assayed for the presence of ASFV in porcine bone marrow (PBM) and peripheral blood mononuclear (PBL) cultures as described above. Samples were assayed at 1∶10 and 1∶100 dilutions, and further dilutions were used if the titre was more than 10^2.0^HAD_50_ or TCID_50_. Serum was collected for antibody detection by indirect ELISA [12]. Pigs that neither developed viraemia nor seroconverted after this period were challenged with a virulent, haemadsorbing tick isolate (OUR T88/1) to confirm that ASFV infection would elicit a clinical or antibody response.

### ASFV detection in cell culture

Primary cultures of pig bone marrow (PBM) cells or pig blood leukocytes (PBL) were used to detect the presence of ASFV by haemadsorption (HAD) or cytopathic effect (CPE) [13], [14]. Live ticks were

killed by freezing at −80°C and the ticks were macerated in pools using Tenbroek tissue grinders. Samples were tested undiluted and at 1∶10 dilution in PBS. Because previous work has suggested that up to three passages in cell culture may be required to detect HAD [15], apparently negative samples were re-introduced into fresh cultures up to three times, after another cycle of freezing and thawing to disrupt cells and increase the release of potential virus. Inconclusive results were confirmed by PCR [16] or direct immunofluorescence (DIF) [17]. The titres of the samples were determined by ten-fold dilution [18] and expressed as log_10_ 50% haemadsorbing doses (HAD_50_) or tissue culture infectious doses (TCID_50_) per ml. Because primary cultures vary in sensitivity, the Malta 78 isolate of ASFV was used as a positive control in these experiments in addition to a negative control.
